# Advances and opportunities in ultrafast X-ray crystallography and ultrafast structural optical crystallography of nuclear and electronic protein dynamics

**DOI:** 10.1063/1.5110685

**Published:** 2019-09-24

**Authors:** Jasper J. van Thor

**Affiliations:** Molecular Biophysics, Imperial College London, London SW7 2AZ, United Kingdom

## Abstract

Both nuclear and electronic dynamics contribute to protein function and need multiple and complementary techniques to reveal their ultrafast structural dynamics response. Real-space information obtained from the measurement of electron density dynamics by X-ray crystallography provides aspects of both, while the molecular physics of coherence parameters and frequency-frequency correlation needs spectroscopy methods. Ultrafast pump-probe applications of protein dynamics in crystals provide real-space information through direct X-ray crystallographic structure analysis or through structural optical crystallographic analysis. A discussion of methods of analysis using ultrafast macromolecular X-ray crystallography and ultrafast nonlinear structural optical crystallography is presented. The current and future high repetition rate capabilities provided by X-ray free electron lasers for ultrafast diffraction studies provide opportunities for optical control and optical selection of nuclear coherence which may develop to access higher frequency dynamics through improvements of sensitivity and time resolution to reveal coherence directly. Specific selection of electronic coherence requires optical probes, which can provide real-space structural information through photoselection of oriented samples and specifically in birefringent crystals. Ultrafast structural optical crystallography of photosynthetic energy transfer has been demonstrated, and the theory of two-dimensional structural optical crystallography has shown a method for accessing the structural selection of electronic coherence.

## I. BACKGROUND

The desire to create “molecular movies” of protein function has driven rapid technological advances in the area of ultrafast crystallography. The definition of a true molecular movie should reference the ultrafast single-molecule dynamics and is not formally applicable to measurements of ensembles such as those that are obtained from ultrafast structural dynamics methods which are discussed in this contribution. Ultrafast X-ray crystallography and ultrafast structural optical crystallography provide access to molecular transformations on the coherent time scale and are both highly selective in their observations. This is the key point and focus of this contribution, to discuss their complementary nature and identify opportunities for future developments. Enabled by the advent of X-ray Free Electron laser (XFEL) sources operating at Angstrom wavelengths and beamline technology, it has become possible to perform femtosecond time resolved pump-probe experiments revealing ultrafast structural dynamics.^1–3^ Now that time resolved X-ray crystallography is possible in the coherent time domain, fundamental questions regarding the control, assignment, and analysis of ultrafast motion have arisen. We must apply lessons learned from decades of ultrafast spectroscopy to this new capability and address the details of the nonlinear optical response and the control and preparation of vibrational coherence. Ultrafast pump-induced differences are generally isomorphous in the case of protein crystals, which allows the generation of Fourier-difference analysis of electron density changes.^1–3^ The ultrafast time scale is not always a requirement for the observation of isomorphous differences following illumination, with many examples from the synchrotron-based and XFEL-based time resolved crystallography with longer pump-probe delays as well.^4–14^

A comparison with the field that studies small molecule X-ray diffraction on ultrafast time scales reveals both fundamental differences and analogies with the macromolecular crystallography methodology. Laser-based hard X-ray diffraction of small molecules has also inspired accelerator-based experiments. Particularly, table-top based ultrafast powder diffraction has been very successful and has optimized and exploited the available flux for measurements of ultrafast nuclear and charge density dynamics.^15–18^ Compelling and recent developments include reports on terahertz driven motion using this technique.^19–21^ Whereas early instruments used near-IR sources for the hard X-ray generation, the brightness is substantially increased with the use of intense mid-infrared pulses generated by an Optical Parametric Chirped-Pulse Amplifier, improving capabilities of table-top sources significantly.^22,23^ The combination of small unit cells, large scattering cross sections, and high spatial resolution has led the small molecule field to advance ahead of macromolecular X-ray crystallography significantly with regard to the measurement of the nuclear coherent response.^24^ We also consider ultrafast electron diffraction methods, which are similarly used to probe small molecule samples, with a number of recent advances in instrumentation and analysis methods reported,^25^ including also gas phase diffraction studies.^26^ Femtosecond time resolved electron diffraction is a powerful technique that is perhaps comparable to table-top laser based hard X-ray sources in certain respects^25^ but currently has lower limits for time resolution and brightness when compared to XFEL sources which could be significantly brighter and deliver shorter pulses.^27^ The experimental requirements for an ultrafast macromolecular X-ray crystallography experiment, which is the topic of discussion for the present contribution, are fundamentally different. The differences include the experimental measurement of pump-induced changes. In the case of powder pattern or single crystal experiments of a small molecule, the Bragg diffraction measurement of pump-induced differences is directly analyzed from the time dependent intensity differences of individual reflections. For macromolecular crystallography, the combination of large unit cell dimensions, light atoms having small cross sections, and often small isomorphous differences dictates the X-ray source requirements.^14,28–32^ The sensitivity to retrieve real-space differences for small molecule X-ray crystallography is aided by direct methods and solvent flattening which improve the phasing and statistics. Typically, for protein crystals, the photoinduced differences are analyzed in real space from the electron density differences obtained by inverse Fourier transform rather than on the level of ΔI/I of individual Bragg diffraction spots.^33^ This requires isomorphicity which is typically maintained on ultrafast time scale in protein crystals. Because the full atomic cross section determines the measurements of protein structural dynamics by X-ray crystallography, it is rapidly dominated by displacements and order-disorder transitions through the analysis of isomorphous differences.

This raises another important question relevant to macromolecular X-ray crystallography: valence electron dynamics could conceivably be revealed indirectly though the measurement of the resulting displacement by X-ray crystallography, which would in many cases overwhelm the underlying electron density differences which are at the origin of the motion. Combining the attosecond time resolution and the subatomic spatial resolution with many additional orders of magnitude of signal-to-noise, such differences might be eventually detected through X-ray crystallography, but unlikely with existing sources. For both small molecules and protein structures, both very high, subatomic, structural resolution, typically better than 0.6–0.8 Å, and very good statistics are needed in order to obtain electron density differences that can be assigned to valence electrons. For example, at 0.66 Å resolution and R-factors better than 10%, charge density analysis of the protein aldose reductase has been presented,^34^ based on the Coppens multipolar model.^35^ Another example of charge density analysis at 0.48 Å resolution also involved a redox protein.^36^ The small molecule crystallography field has many more examples of charge density analysis at subatomic resolution, and the field of “photocrystallography” includes time resolved work conducted at synchrotron sources.^37,38^

Even if subatomic resolution femtosecond X-ray crystallography could reveal the extremely small electron density differences of, for example, the molecular (Frenkel) exciton dynamics in light harvesting complexes, there are numerous physical processes that still need to be revealed by spectroscopy methods. Ideally, a structural sensitivity for ultrafast spectroscopy is obtained.

Therefore, ultrafast spectroscopy offers a complementary and necessary methodology to probe the valence electron dynamics and coherence. In order to add a structural sensitivity in the molecular frame, ultrafast spectroscopy measurements of oriented single crystals have recently been successfully demonstrated.^39^ An exciting prospect is that methods developed by the nonlinear spectroscopy community, when applied to oriented single crystals, should likewise yield a real-space analysis of coherences.^40^ The two different techniques of ultrafast X-ray crystallography and ultrafast optical crystallography measure different aspects of structural dynamics but are married through the necessary application of crystal spectroscopy and crystal optics analysis in both cases.^40,41^

This contribution aims to bridge the communities of ultrafast spectroscopy and ultrafast crystallography, with an emphasis on protein dynamics. Inspiration is additionally taken from the small molecule ultrafast X-ray diffraction community which is extremely useful to compare with experimental capabilities for protein crystallography. The ultrafast protein X-ray crystallography capabilities have been recently developed and represent an emerging field in ultrafast structural dynamics. Section II is provided to give an introduction and discussion of the technical background of the measurement of time resolved X-ray crystallographic differences and considers future capabilities with high-repetition rate instruments. The section thus gives an overview and introduction that is useful to the ultrafast spectroscopy community as well. Section III collects a number of technical aspects specific to the high intensity excitation used for ultrafast crystallography, and it explains and summarizes how methods that have been taken from ultrafast spectroscopy can be used for the analysis and control of structural dynamics observed in ultrafast X-ray crystallography. Section IV emphasizes methods of structural analysis which are available for spectroscopy techniques, specifically the opportunities that arise from making single crystal optical measurements. The results are additionally directly relevant to the previous sections on X-ray crystallography as they connect the optical control of coherences and populations with structural dynamics observation.

## II. X-RAY SOURCE PARAMETERS AND CRYSTALLOGRAPHIC SIGNAL-TO-NOISE FOR STATIONARY APPLICATIONS OF TIME RESOLVED X-RAY CRYSTALLOGRAPHY

Emerging ultrafast capabilities for protein X-ray crystallography at XFELs offer new opportunities to probe nuclear coherence and dynamics. Here, two pertinent questions are addressed. First, methods of optical control and calculation are discussed to analyze and assign ground state and excited state coherences on the ultrafast time scale. Second, future capabilities including high repetition rate or multipulse noncollinear methods could develop the signal-to-noise of the X-ray crystallographic measurement of light induced differences in order to reveal as of yet hidden aspects of molecular dynamics.

The general field of time resolved protein X-ray crystallography, established by Moffat and colleagues in the 1990s,^5–12^ has recently developed to allow measurements with femtosecond pump-probe delays.^1–3^ These breakthrough demonstrations have been possible through the development of hard XFEL sources and beamline instrumentation to allow pulsed application of crystallography. Protein crystallography at XFELs has been made possible from the application and analysis of serial diffraction methods of still images. Early analysis of single crystal datasets collected at the Linac Coherent Light Source (LCLS) XFEL using quasirotation data collection provided a quantitative analysis of the structure factor amplitude noise and sensitivity under representative conditions using Self-Amplified Spontaneous Emission (SASE) radiation.^42^ XFEL radiation that is generated under SASE conditions has a significant spectral width, typically about ∼40 eV width, a sharp and fluctuating spectral distribution, and jitter of the mean photon energy. For X-ray crystallographic measurements, this results in significant uncertainty not only of the partiality arising from the stationary geometry but also from the unknown wavelength normalization. The experimental results highlighted the poor statistics and partiality caused by the SASE radiation crystallography of stationary crystals, even in quasirotation mode. This analysis indicated that the signal-to-noise required for time resolved applications that must resolve very small amplitude differences is limited by source intensity and spectral noise, spatial and mode fluctuations, mosaic spread, and number of observations.^42^ Particularly, the latter has been addressed in the serial femtosecond crystallography (SFX) method, which uses thousands of indexed diffraction images to retrieve accurate mean structure factor amplitudes through extensive averaging. The earliest demonstrations of meaningful structure factor amplitude information from serial femtosecond crystallography retrieved ΔI/I signals on the order of ∼20% suitable for *ab initio* structure solution by the anomalous dispersion method.^43^ Briefly after, flash-induced differences were demonstrated using the serial crystallography technique, which relied on very high conversion by intense nanosecond optical flashes.^44^ Recent developments have shown significant promise for including postrefinement to further improve the quality of the retrieved amplitudes.^45–48^ Such improvements have increased the data quality and crystallographic statistics from the serial crystallography data and aid the ability to detect small photo-induced differences. The sensitivity to detect pump-probe differences is a key consideration for current and future applications for ultrafast X-ray crystallography. Note that it is not necessary to resolve pump-induced ΔI/I differences better than noise, as the inverse Fourier transform in well correlated data accumulates electron density differences in real space to levels of statistical significance. Nevertheless, the present capabilities and available repetition rates at XFEL beamlines place limits on the number of merged frames in time resolved datasets. At the European XFEL^32^ and the future LCLS-II, kilohertz-megahertz repetition rates will significantly increase the available signal-to-noise. As a rule, in order to successfully measure photo-induced differences under optimal conditions for SFX, it is, however, currently necessary to achieve at least 10% conversion of the crystals. This requirement must also be weighted by the magnitude of real-space displacement and is furthermore a function of the scattering cross section of the elements involved.

The time resolved application of serial femtosecond crystallography is significantly aided by the single-shot nature of the experiment. In comparison, synchrotron based pump-probe experiments, even with bright dual undulator pink beams such as APS/14ID BioCARS operated in hybrid mode, typically accumulate Bragg diffraction intensity on the detector to fill the dynamic range from multiple pump-probe cycles. Also, with successful single exposures such as shown for myoglobin-CO, the same crystal volume is typically repeatedly exposed following rotation for the data collection.^49^ The resulting accumulating damage from both the X-ray exposure and the laser exposure can rapidly degrade diffraction quality as well as generate difference structure factor amplitude that is unrelated to the photoreaction of interest. Such modification and background can rapidly overpower the desired pump-induced structure factor amplitude differences. The alternative for time resolved synchrotron-based Laue crystallography applies serial crystallography, often in combination with optical excitation, in combination with CW mode probes. In this case, the megahertz fill structure is maintained rather than using hybrid or single high-current mode, and millisecond exposures collect still images from stationary targets.^50^ The time resolution for this application is therefore milliseconds, and while good results have been obtained, there is the open question of significant sample heating even under native conditions.^51^

**FIG. 1.**
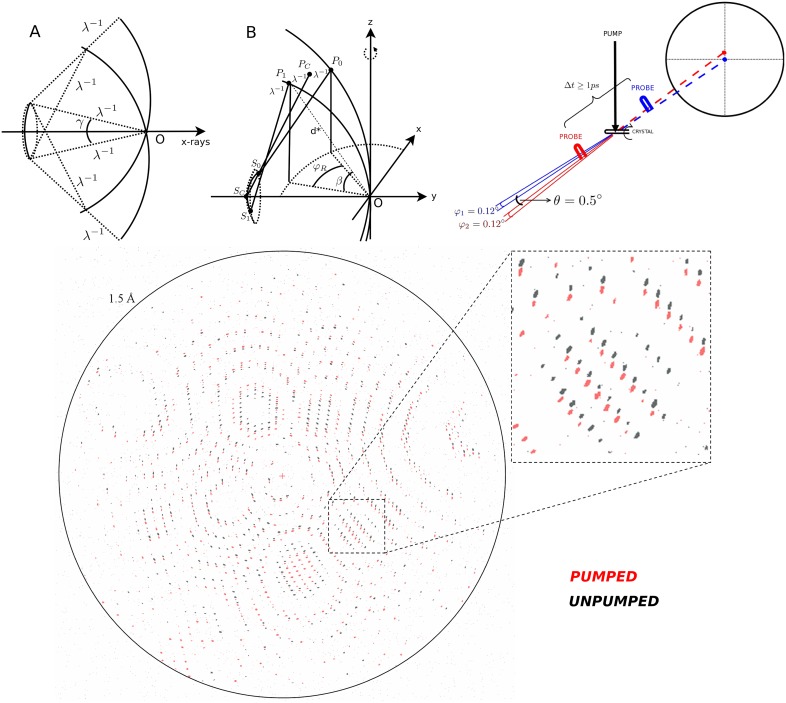
A possibility for achieving orders of magnitude enhancement for the experimental measurement of photoinduced structure factor amplitude differences. A proposed noncollinear three-pulse probe-pump-probe experiment, combined with the convergent-beam method, may be combined with a high-dynamic range transmissive intensity measurement of both focused X-ray beams for additional intensity normalization. The figure shows Ewald construction, beam geometry, and a stationary simulation based on realistic and achievable parameters for a split-and-delay line together with CRL-array focusing. Reproduced with permission from van Thor and Madsen, Struct. Dyn. **2**, 014102 (2015). Copyright 2015 AIP Publishing.^56^

While the single-shot configuration of the XFEL based experiment ameliorates concerns that apply to the synchrotron geometry, there are also a number of disadvantages relative to the synchrotron case. Spectral fluctuations and intensity noise are significant especially in SASE operation,^42^ while the expected data quality improvements from using self-seeded mode have not been strongly demonstrated.^52^ Different approaches to postrefinement, and reported successes in the improvement of statistics, to some extent ameliorate the problem of partiality arising from the Monte Carlo result from serial crystallography.^48,53^ Typically, a mean photon energy is measured in SASE mode which is used for crystallographic processing as well. Clearly, crystal quality, crystal mosaic spread, isomorphicity, twinning, crystal morphology, and homogeneity all contribute to the final merging statistics and their specific response to X-ray beam parameters. Nevertheless, it is now commonly seen that in the case of well diffracting crystals, perhaps 5 K or 10 K images depending on symmetry may be merged to successfully reveal photo-induced differences depending on the magnitude of photoconversion. This very good news means that it is now feasible to collect multiple pump-probe time delays for time resolved measurements using the time-resolved serial femtosecond crystallography (TR-SFX) method from a single beamtime at instruments that operate at relatively low repetition rates and are not superconducting, such as SPring-8 Angstrom Compact free electron LAser, LCLS, and SwissFEL.^44,54,55^ These considerations also guide and shape our expectations for new science that will be possible at future high repetition rate XFELs. While the LCLS-II will be able to deliver a 1 MHz rate, it is likely that data throughput will be significantly limited by the detector frame rate. X-ray detector technology must be significantly developed to exploit the repetition rate improvement of megahertz XFEL sources. Nevertheless, megahertz repetition rate sample delivery has now been achieved.^32^ It is clear that LCLS-II will deliver a significant step-change, resulting in crystallographic signal-to-noise from the increased repetition rate. With this improvement, it is expected that our ability to detect much smaller populations and also smaller real-space displacements will increase. This exciting prospect could be exploited to pursue and reveal new aspects of structural dynamics and particularly to advance the time resolution to the few-femtosecond limit and to create the ability to perform a measurement of high frequency vibrational coherence from the X-ray crystallographic equivalent of an impulsive time domain Raman measurement, for which the theoretical considerations will be reviewed below. In addition to future high repetition rate applications, alternative proposals exist for improving the accuracy of the measurement of photoinduced structure factor amplitude differences^56^ (Fig. 1). A ratiometric measurement has been proposed which aims to achieve the simultaneous measurement of the pumped and unpumped diffraction from a single crystal volume by obtaining a spatial separation from a noncollinear beam geometry. A convergent geometry for the stationary case will additionally ameliorate any inaccuracy of partiality determination (Fig. 1).^56^ Furthermore, it would be of significant advantage to include transmissive intensity measurements of both probe and reference beams with a high dynamic range in order to address the stability performance of a split-and-delay instrument.^56^ A further requirement would be that the first interaction avoids X-ray induced destruction such that the second X-ray pulse still probes the crystal volume. A compromise for such measurements between the probed crystal volume, X-ray flux, and photolysed population should likely be intermediate between the micrometer sized crystals typically used for serial femtosecond crystallography (SFX) and attenuated conditions used for single crystals corresponding to the typical synchrotron geometry.^56^ Alternatively, the noncollinear scheme shown in Fig. 1 could be applied to high-flux destructive TR-SFX conditions with a small few-micrometer spatial offset of focus of the two beams. In this geometry, there would still be very high correlation possible between pumped and unpumped diffraction images, while still exploiting the diffract-before-destruct regime. While such an instrument would rely on a high performance split-and-delay line,^57^ further instrumentation including microarrays of compound refractive lenses (CRLs) would also be needed for implementation. Nevertheless, with the potential to achieve orders of magnitude enhancement of difference measurement, this would be a route to develop the ability to measure nuclear coherence for protein crystallography specifically using low- or intermediate repetition rate accelerator technology.

An obvious alternative to applying the convergent beam method to increase partiality is the application of Laue crystallography. Whereas the typical SASE bandwidth for hard X-ray radiation at XFELs is typically ∼0.1%–0.2% ΔE/E, at the SwissFEL, a large bandwidth mode has been developed that can generate pulses with an ∼4% ΔE/E bandwidth.^58^ The scheme relies on overcompression of the electron bunch to allow wakefield generation of an energy chirped bunch. Recent demonstration at 6 keV showed a 2% chirp which generated a 4% bandwidth that could be used for Laue crystallography in serial femtosecond crystallography mode. The advantage of collecting Laue data is that full partiality may be obtained, while an energy gradient across the reflections should still be quantified. Such data may be analyzed using traditional Laue crystallography methods. The large bandwidth mode at SwissFEL also raises the intriguing possibility to obtain ultrafast time resolution in q-space from the energy to time mapping that is intrinsic in the chirped pulse generation. Since different reflections are stimulated by different energies, there is a correspondence to different pump-probe timings for each reflection in the time resolved application. This is additionally true for the energy gradient that is present across each reflection. Moffat first described the principle of exploiting an energy-to-time mapping and proposed the spontaneous generation of chirped X-rays at synchrotron sources through electron bunch chirping in 2002.^9^ The possibility of an ultrafast application from XFEL chirped Laue crystallography would follow similar considerations for the retrieval of temporal information. A theoretical modeling of the achievable time resolution in chirped Laue mode would additionally need to include a detailed physical description of cross correlation.^59^

## III. THE CONSEQUENCES OF INTENSE FEMTOSECOND OPTICAL EXCITATION REQUIRED FOR SUB-KILOHERTZ REPETITION RATE TIME RESOLVED PROTEIN X-RAY CRYSTALLOGRAPHY

With currently available repetition rates, ultrafast time resolved protein X-ray crystallography experiments are conducted under excitation intensities that are potentially inappropriate unless careful characterization and optimization established linear and nonlinear processes. On the other hand, opportunities for optical control of molecular dynamics can exploit the high power regime.

As outlined above in Sec. II, the necessity to generate a significant fraction of photoexcited population to create detectable difference electron density signals requires the use of very intense visible laser pulses. The problem is the following: we wish to show and measure the behavior of light sensitive materials under conditions that are typically the weak incoherent illumination of the sun. However, in order to achieve the ultrafast time resolution, we are required to use extremely intense pulsed excitation, or no signal will be measured. This is the case for the currently available repetition rate and detector bandwidths. Potentially, in the future, we may be able to detect negligible populations under representative illumination, but the data rate required would be difficult to predict, if it will be possible at all. To put the illumination intensities into perspective, a Solar Constant (one sun), as defined by the World Metrological Organization, is 1.367 mW/mm^2^.^60^ However, the illumination of a photoreceptor protein crystal for the execution of ultrafast X-ray crystallography applied an intensity of 1 GW/mm^2^, albeit for a short 150 fs duration.^2,33,41,61,62^ The peak power that is used is thus ∼7 × 10^11^ times higher than conditions under the sun.

There are a number of consequences resulting from such intense illumination. First, nonlinear multiphoton reactions are likely driven under such conditions, including photoionization events^63–66^ such that the resulting structural dynamics are not necessarily representative of the biological function. The need to carefully control and characterize the multiphoton processes may only be overcome with significantly increased sensitivity of the X-ray crystallographic measurement such that very small populations may be prepared and probed (see Sec. II).

**FIG. 2.**
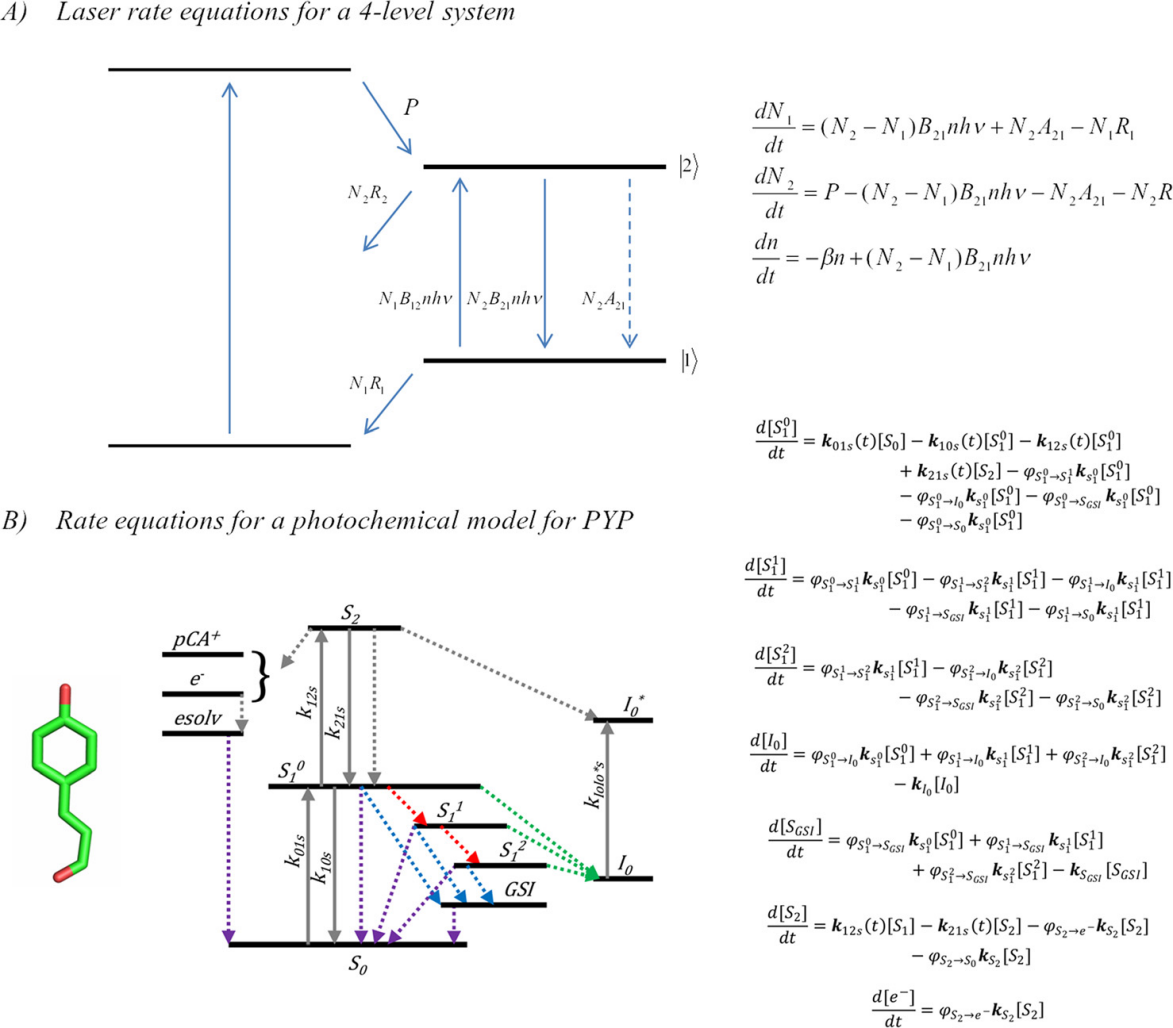
Illustration of the use of laser rate equations for the analysis of time and photon flux dependence of population transfer under intense femtosecond illumination of protein crystals. (a) The conventional illustration for a 4-level laser uses incoherent laser rate equations under conditions of a pumping rate P, population densities N_i_, spontaneous emission probability Ai, relaxation rates R_i_N_i_, and stimulated absorption end emission rates proportional to the Einstein coefficients B_ik_. (b) Coupled differential rate equations used to model the Photoactive Yellow Protein reactions from femtosecond transient absorption spectroscopy. (Inset) Reproduced with permission from Lincoln *et al.*, Phys. Chem. Chem. Phys. **14**, 15752 (2012). Copyright 2012 Royal Society of Chemistry.^61^

At the time of writing, there is one existing example where the reported ultrafast protein crystallography experiments have included the explicit analysis of nonlinear transformations, including in crystals, which are the ultrafast crystallography measurements of the Photoactive Yellow Protein (PYP).^2,33,41,61,62^ Early systematic studies of the PYP evaluated the dependence on the peak power, pulse duration, carrier frequency, and second order dispersion on the phototransformation yields and effective cross sections obtained from target analysis.^61^ The objective of the study was to use incoherent models to extract the explicit product amplitudes and nonlinear cross sections using published models for the photochemical and thermal transformations, which needed model-independent verification. Figure 2 shows that such analysis is essentially equivalent to using incoherent laser rate equations and involves the construction of a set of coupled differential equations. Thus, a simultaneous global target analysis of a series of transient absorption measurements made with systematic variation of optical parameters retrieved the explicit yields and corresponding spectral differences, which relied on known spectral assignments.^61^ The latter also separated the different multiphoton products spectrally, which in the case of PYP includes photoionization and radical formation in addition to excited state absorption and stimulated emission. However, a model-free analysis may also evaluate and quantify the total accumulated nonlinear cross section from essentially an adaptation of the open-aperture Z-scan experiment. When applied to samples of PYP, the model-free results from Z-scan experiments corresponded well with the results from the transient absorption analysis.^61^ While the simultaneous global analysis method may be elaborate and time consuming, the Z-scan method is much simpler in execution and can provide considerable confidence to test and optimize optical parameters suitable for pump-probe crystallography applications. The additional advantage is that such Z-scan methodology may be more readily applied to crystal measurements, which has been shown for PYP.^2,62^ Ultrafast TR-SFX experiments of PYP were conducted under carefully optimized and controlled conditions with regard to peak power, pulse duration, magnitude and sign, second order dispersion, and carrier frequency for which both optical measurements and X-ray crystallographic characterization showed that linear processes dominated and product yields in crystals exceeded 10%.^2,62^

There is not a specific and general upper limit to the optical power density that may be applied for ultrafast TR-SFX in order to avoid nonlinear processes, as this is strongly dependent on the system as well as the optical parameters. From excitation studies of heme proteins, Miller concludes that optical power density should not exceed 100–200 GW/cm^2^ under resonant conditions.^67^ This conclusion is specific for heme proteins, where indeed transient absorption under representative conditions has shown a very large excited state cross section, such that the ultrafast induced absorption exceeds the ground state bleaching in many conditions.^68,69^ Under such conditions, it is almost unavoidable to drive multiphoton processes under resonant conditions, and acceptable power density would indeed typically be limited to the 100 GW/cm^2^ level or less. For the possible situation that at the laser carrier frequency and bandwidth used, the excited state cross section is negligible or absent, femtosecond excitation could drive significant ground state bleaching avoiding multiphoton processes; in a two-level system, the population could theoretically reach 50% and multiphoton processes would proceed through Rabi cycling. Systematic studies have demonstrated the very strong differences in nonlinear transformation from power density titrations depending on optical parameters.^2,33,41,61,62^ For instance, 400 nm excitation of PYP using 300 fs stretched pulses having ∼7000 fs^2^ second order dispersion estimated contributions for S2-products to be less than 2% at 15.4 GW/cm^2^, but limiting ground state bleaching to 5%. At a much higher intensity of 138 GW/cm^2^, the S2-products involving excited state absorption had overtaken the “productive” S1-products.^61^ This demonstrates that even stretching is ineffective to avoid excited state absorption with 400 nm excitation, and at low power, the isomerization yield, taken as the product of 0.05 and the primary quantum yield would be estimated at 1% when avoiding S2-reactions.^61^ Similarly, pumping at 490 nm, irrespective of stretching, limits photoisomerization from the very efficient stimulated emission pumping. At that wavelength, the excited state cross section significantly exceeds the ground state cross section and less than 3% ground state bleach may be generated that avoids significant stimulated emission pumping, yielding approximately 0.6% isomerization, while intense pumping at 300 GW/cm^2^ only further decreases this yield. This situation is in fact equivalent to the heme protein case, where excited state cross section exceeds the ground state cross section, typically limiting femtosecond excitation to less than 100 GW/cm^2^. For PYP, an optimal condition has been identified with direct on-resonance excitation at 450 nm, which showed some, but controllable, excited state cross section which was significantly less than the ground state cross section. Avoiding both excited state absorption and stimulated emission, moderately stretched 140 fs pulses maintained a 4-fold excess of S1-processes at powers exceeding 60 GW/cm^2^.^61^ This was further confirmed from optical measurements made on crystals of PYP, which showed a stronger dependence on the pulse duration and second order dispersion as compared to solutions but identified acceptable power density between 100 and 200 GW/cm^2^.^62^ It has also been shown that an excitation of 100 GW/cm^2^ or more causes significant nonresonant 3-photon ionization of water.^70^ It remains to be determined how such nonresonant processes would contribute to time resolved protein X-ray crystallography measurements. Focusing on resonant excitation conditions, the example of PYP has demonstrated methods of measurement and analysis that allow the execution of ultrafast X-ray crystallography under conditions of defined and quantified optical conversion.^2,33,41,61,62^ Furthermore, femtosecond spectroscopy investigations are often conducted on dilute solutions of proteins which may not be representative for the crystalline environment. Protein crystals are more viscous, and it is often found that quantum yields, femtosecond kinetics, branching ratios, and other parameters are modified relative to the dilute material. This necessitates optical measurements on crystalline protein materials for the practical purpose to verify and design ultrafast X-ray crystallography conditions, which has been demonstrated for the Photoactive Yellow Protein.^62^ In this regard, it is of interest to note that coherent control experiments have suggested that vibrational dephasing which was controlled by solvent viscosity is the main limiting factor for control in an ensemble.^71^ Furthermore, structural differences for constrained proteins in the lattice relative to dilute solutions may account for differences of photochemical properties as well. Whether detectable populations can be reached in order to conduct an ultrafast TR-SFX experiment successfully are very much dependent on the specific magnitude of the effective optical cross sections, and this may prevent some materials to be used with current signal-to-noise available at XFELs.

**FIG. 3.**
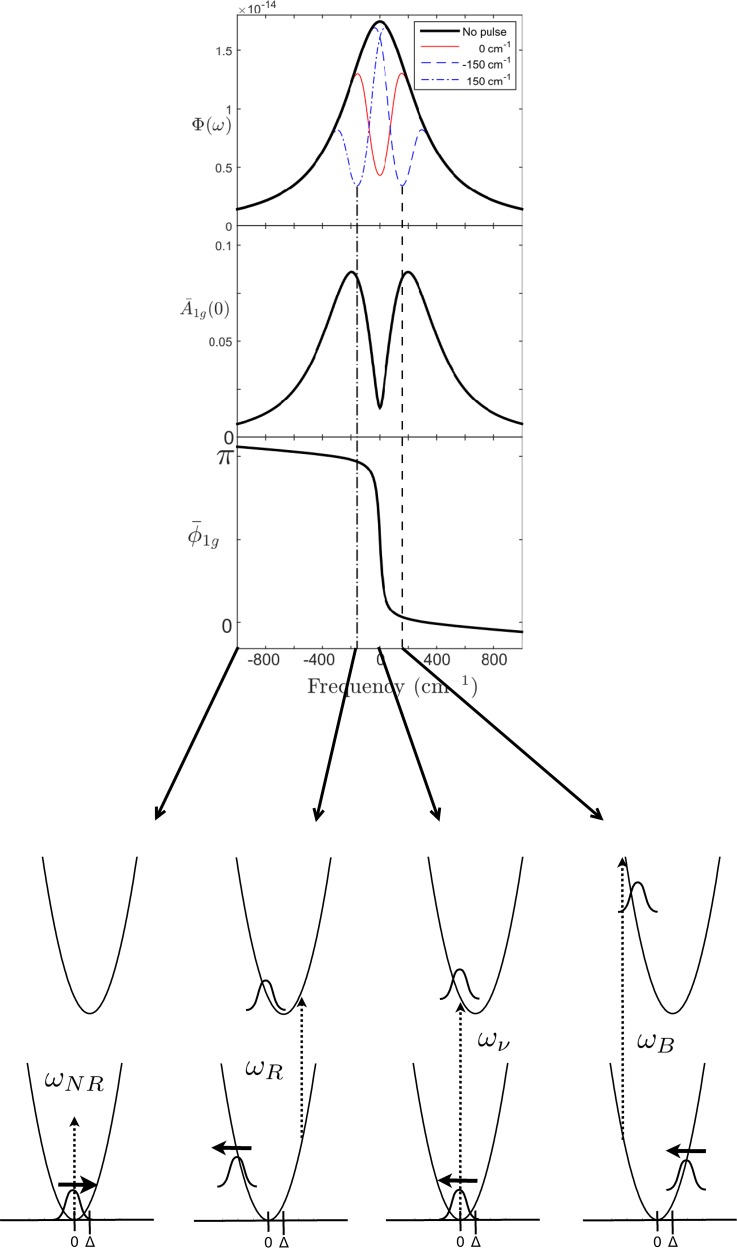
Illustration of the line shape theory treatment of ground and excited state vibrational coherence calculation using additional wavepacket representations for a high temperature case (300 K). Laser excitation of a homogeneously broadened line Φ(ω) with carrier frequency below (ω_R_), on (ω_ν_), or above (ω_B_) resonance creates a total impulsive ground state coherence A_1g_ with phase φ_1g_. The resulting ground coherence is shown for the high temperature case including arrows for the direction of the imparted impulse momentum as shown by Kumar *et al.*^72,73^ The amplitude of ground state coherence for the nonresonant (NR) case is orders of magnitude below the resonant conditions.^72,73^ (Top of figure) Reproduced with permission from Hutchison and van Thor, Philos. Trans. A **377**, 2145 (2019). Copyright 2019 The Royal Society.^41^

A second consequence of the use of intense femtosecond optical excitation is that typical pulse durations and optical bandwidths such as those selected for the PYP create two types of vibrational coherences (Fig. 3). Typically, the laser spectrum is narrower than the inhomogeneously broadened absorption bands of light sensitive proteins, such that electronic excitation is selective for specific nuclear coordinates according to their Franck-Condon integrals. The time-bandwidth product determines the frequency limit for ground state vibrational coherence that results from the nonequilibrium geometry that is prepared under such conditions and is impulsive in nature. Under conditions of resonance or near-resonance, the direction of the imparted momentum is in the direction of the nuclear binding force (Fig. 3). Moreover, the magnitude of the ground state vibrational coherence is additionally determined by the population and may thus be controlled by the laser power and spectrum.

Excited state vibrational coherence is not generated impulsively and is displacement driven, and the photolysed population weighs the ensemble contribution to the motion that is observed. The pulse duration additionally sets the limiting frequency for the coherent motions.

Kumar *et al.* made the generalization that for short lived excited states and for off-resonant, or near-resonant, conditions, ground state coherence will dominate the impulsive Raman measurement, whereas for long lived excited state and weak excitation conditions, the excited state coherence may dominate.^72,73^

The inescapable conclusion, supported by the results from decades of time domain Raman spectroscopy research, is that for current applications of femtosecond time resolved pump-probe X-ray crystallography, both ground state and excited state vibrational coherences are generated with significant amplitudes.^41,62^ From impulsive Raman spectroscopy, it is known that with under intense femtosecond optical excitation with a moderate laser bandwidth, approximately half of the motion that occurs within the vibrational dephasing time is due to ground state coherence, which is unrelated to the reaction coordinate that is usually of biological interest.^41,62^ Realistic experimental bandwidths reported are on the order of ∼3 THz,^1,2^ and vibrational dephasing times in proteins are on the order of 1–2 ps.^72–76^ Therefore, the first few picosecond pump-probe delays must be analyzed for the contributions of vibrational coherences. Taking established results from the ultrafast spectroscopy field, experimental and theory methods can be applied for such analysis of ultrafast TR-SFX. In particular, explicit line shape analysis should provide an intuitive and powerful approach to experimentally control vibrational coherence. The established representation using the “Wigner phase space”^77–79^ is most useful as it provides the classical parameters, the complex coherence amplitude, $|A_{1g}|=\sqrt{\left(Q_{g}^{2}(0)+P_{g}^{2}(0)\right)}$, position Q_g_, phase $\Phi_{g}=-{\text{tan}}^{-1}\left[\frac{P_{g0}}{Q_{g0}}\right],$ and impulse P_g_. As briefly revisited below, line shape theory treatment of a two-level problem is performed in approximation using cumulant expansion techniques. In addition to direct pump-induced coherence in ground and excited states, fast nonradiative processes can also generate vibrational coherence in product states. Adiabatic Landau-Zener transitions involving an excited state crossover can leave a vibrationally coherent product state, as has been demonstrated in early experiments with rhopdopsin.^80^ Both direct laser driven and Landau-Zener coherence have been treated in coherence theory.^72,73,81^

Other methods in the separated pulse limit have used the calculation of nonstationary effective response functions based on the third order response formalism.^82–84^ It has been pointed out that in the third-order formalism of pump-probe dynamics, there is not a clear separation of the pump and probe responses, while the line shape-function analysis that provides Wigner phase space results developed by Kumar *et al.* is particularly useful for obtaining this separation.^72,73,81^ An important correction additionally arose from the development of Wigner phase space representation of the pump-probe interactions; in prior linear response function treatment, it was concluded that the ground state wave packet is always created on the side of the well that is closest to the excited state potential minimum.^82,83,85^ Kumar *et al.*, however, showed that the ground state coherence is in fact strongly dependent on the carrier frequency, which is illustrated in Fig. 3. It is this conclusion and quantifiable results that will provide experimental means by which the femtosecond dynamics from TR-SFX experiments can be controlled and analyzed.

Future experiments will be able to use the results of coherence calculations in order to predict and control the femtosecond dynamics by optical means. In practice, active pulse shaping techniques using an acousto-optic programmable dispersive filter (AOPDF) will be helpful to achieve this at XFEL beamline stations that will allow computer programmable control and shot-to-shot capabilities.

The Raman spectroscopy field has analyzed the time-domain measurements of vibrational coherences using both experimental and theoretical approaches with the goal of separating and assigning the ground and excited state contributions.^72,73,76,82,83,85–93^ A Wigner phase space method was shown by Kumar *et al.* which has the significant advantage that line shape theory allows an intuitive interpretation that through the Kramers–Kronig relationship is additionally applicable to experimental line shapes. Their work uses a cumulant expansion of the density matrix for a two-level system where the first moment is taken to write the Wigner phase space parameters—full details are given in Refs. 72 and 73. A brief summary of the conclusions is relevant primarily because modification is needed because the results are formally an approximation valid only in the weak excitation limit and additionally describe a two-level system only. With regard to the first limitation, the photolysed population which appears in the Wigner phase space calculations can be modified to yield an approximation with intense excitation.^33^ Furthermore, the two-level system problem definition formally prevents population inversion at high intensity. With these caveats, nevertheless, a calculation can be performed that can provide a prediction of the results of optical control in an intuitive manner. In this form, the initial wavepacket position ${\bar{Q}}_{g}\left(0\right)$ of the ground state is given as

Q¯g0=−μge2E022n¯+1Δ8πℏ2Ng×∫0∞dωG~pω−ωc,−ω0Δ^ΦIω, (1)

with the initial impulse momentum

P¯g0=μge2E02Δ8πℏ2Ng∫0∞dω G~pω−ωc,−ω0Δ^ΦRω (2)

and with the product spectral function

$${\tilde{G}}_{p}\left(\omega ,n\omega_{0}\right)=\tilde{G}\left(\omega \right)\tilde{G}\left(\omega +n\omega_{0}\right).$$ (3)

In the weak field limit, the population N_g_ that appears in Eqs. (1)–(3) is found as

$$N_{g}=1-\frac{{\left|\mu_{\mathrm{ge}}\right|}^{2}}{\pi \hbar^{2}}\int_{-\infty }^{\infty }d\omega {\left|\tilde{E}\left(\omega \right)\right|}^{2}\Phi_{I}\left(\omega \right),$$ (4)

with the imaginary part of the complex line shape function

$$\Phi_{I}\left(\omega \right)=i\int_{0}^{\infty }\mathrm{ds}\;e^{i\left(\omega -\Omega_{00}\right)s}e^{-{Г}_{\mathrm{eg}}\left|s\right|}e^{-g\left(s\right)}.$$ (5)

For intense excitation, the expressions for the calculation of the ground state population N_g_ and excited state population N_e_ have been proposed as follows:^33^

$$N_{g}=\exp\left(-\frac{{\left|\mu_{\mathrm{ge}}\right|}^{2}E_{0}^{2}}{4\pi \hbar^{2}}\int_{0}^{\infty }d\omega \;{\tilde{G}}^{2}\left(\omega -\omega_{c}\right)\Phi_{I}\left(\omega \right)\right),$$ (6)

$$N_{e}=1-N_{g}=1-\exp\left(-\frac{{\left|\mu_{\mathrm{ge}}\right|}^{2}E_{0}^{2}}{4\pi \hbar^{2}}\int_{0}^{\infty }d\omega \;{\tilde{G}}^{2}\left(\omega -\omega_{c}\right)\Phi_{I}\left(\omega \right)\right).$$ (7)

Using Eqs. (6) and (7), Fig. 3 (top) illustrates the approximation of the effective “hole-burning” into the standard line shape function using strong excitation with blue (B), red (R), and direct resonant (ν) excitation. The resulting calculations show that, in agreement with weak-field results by Kumar *et al.*,^72,73^ at resonance, the total ground state coherence is minimized relative to the red and blue excitation cases (Fig. 3, middle graph representing the detuning frequency dependence of |A_1g_|.) The result additionally predicts that changing the carrier frequency from blue to red excitation, the phase φ_1g_ of the ground state coherence wavepacket changes from π to close to zero (Fig. 3, bottom graph). This directly guides a possible experiment to modify the phase of ground state coherent motion, which could be controlled by AOPDF with broadband input. With an interleaved shot-to-shot modulation, the coherences could be selectively identified from femtosecond time resolved observation. Similarly, the power density and pulse duration may control the excited state and ground state coherence contributions and properties. Equations (1), (2), and (4) also demonstrate that, in accordance with conclusions from the Raman spectroscopy field, very weak excitation will selectively suppress the ground state coherence through maximizing the population N_g_, whereas the exited state coherence can dominate the pump-probe differences as these are displacement driven. This provides compelling motivation for conducting femtosecond time resolved TR-SFX experiments at megahertz repetition rates with weak excitation and enhanced signal-to-noise that should detect excited state selectively using future generation sources and fast detectors.

**FIG. 4.**
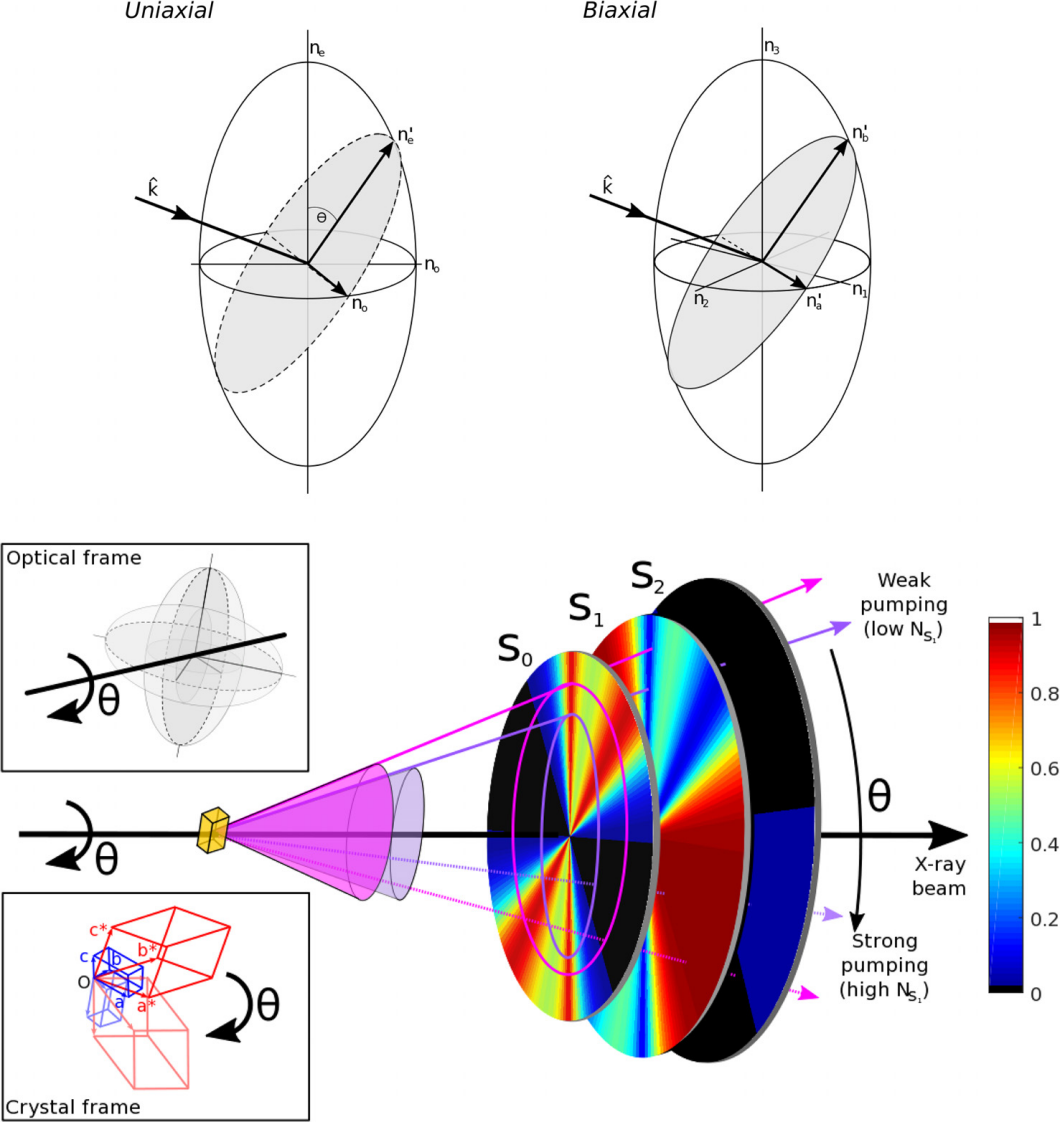
Illustration of the analysis of orientation dependence of photoinduced populations with reference to both the optical orientation and the X-ray crystallographic orientation. The general quadratic representation of uniaxial and biaxial crystal symmetries is ellipsoids of revolution and triaxial ellipsoids, respectively. These allow finding the polarization directions for each k-vector. Tracing a Debye-Scherrer ring in revolution, the polarization directions are modified according to the solutions given by the quadratic representation. Therefore, population transfer may be analyzed radially in the detector plane, as illustrated with a physical calculation example for a uniaxial case. Reproduced with permission from Hutchison and van Thor, Philos. Trans. A **377**, 2145 (2019). Copyright 2019 The Royal Society.^41^

An as yet unexploited opportunity for analysis of pump-induced differences, which is achievable with current experimental capabilities at XFEL stations, is to analyze the orientation dependence of optical pumping.^41^ Stationary laser excitation of a molecular crystal generally results in significant photoselection. Optical crystals are either isotropic (cubic symmetry), uniaxial (trigonal, tetragonal, or hexagonal symmetry), or biaxial (triclinic, monoclinic, or orthorhombic). For all orientations of isotropic crystals and in the optic axis directions of uniaxial and biaxial crystals, double refraction does not occur and a photoselection can be evaluated directly from the indexed crystal orientation provided that there is knowledge of the transition dipole moment direction in the asymmetric unit. In all other cases and assuming transparency, birefringence will produce two orthogonal polarizations which prepare a sum of two field-dipole interactions. The directions of these fields are given by the solutions to Maxwell's polarized wave equations and are in practice found either by evaluating the indicatrix or quadratic representation, method or the wave-surface method which are covered elsewhere.^40,41^ In the presence of birefringence, the field decomposes as

$$E\left(z\right)={\hat{e}}_{1}E_{1}e^{-ik_{1}z}+{\hat{e}}_{2}E_{2}e^{-ik_{2}z},$$ (8)

$$k_{1}=\omega \sqrt{\mu_{o}\epsilon_{1}}=k_{0}n_{e1},\;k_{2}=\omega \sqrt{\mu_{o}\epsilon_{2}}=k_{0}n_{e2},$$ (9)

where $E_{1}$ and $E_{2}$ are the amplitudes of the fields of in ${\hat{e}}_{1}$& ${\hat{e}}_{2}$ and n_e1_ and n_e2_ are the two refractive indices. As a consequence, the measured response becomes the sum of the two separate field-dipole interactions, which significantly modifies the measurement relative to isotropic materials.^39,94–96^ The phase velocity difference between two modes is small or even negligible for small microcrystals but should maximize at the anomalous dispersion region near resonance.^33^ Experimentally, the intrinsic birefringence should be known exactly in order to calculate the polarization directions in the presence of birefringence. The crystal morphology variations and resulting refraction will add uncertainty to the polarization retrieval which is performed for propagation inside the crystal medium, but in principle, the analysis is possible by obtaining the crystal orientation from inversion of the indexing matrix.^41^ Following the polarization calculation, a conventional calculation of linear and nonlinear conversion can be performed,^41^ equivalent to the isotropic analysis of power titration analysis of transient absorption.^33,61^ In this approach, the retrieval of linear and nonlinear cross sections obtained from the power density variation results in modeling of photochemical populations. The connection between crystal orientation, X-ray diffraction, and phototransformation is shown in Fig. 4.^41^ Practically, a pump-probe TR-SFX experiment would need to collect many more stationary diffraction images in order to add orientational analysis. For each pump-probe delay, binning of frames according to computed population would allow an experimental separation of the linear and nonlinear contributions in the difference electron density. This proposed method would therefore also significantly benefit from future high repetition rate XFEL sources.

This analysis presents a significant opportunity to also analyze already existing XFEL crystallography data because currently, the full Debye-Scherrer ring for each reflection is scaled to the same intensity. Therefore, the existing analysis averages the differences in photolysed contributions, and Fig. 4 shows the principle of separation based on crystal optics and X-ray crystallographic indexing. A more complicated case would present itself if more than one dipole was present in the asymmetric unit, and coupling exists between them. In that case, the theoretical calculation has shown that depending on the crystal orientation and the polarization that is prepared, particular coherences and Liouville pathways become symmetry disallowed.^40^ This has been calculated and shown for four-wave mixing conditions but is also true for the interaction of rank of two that is sufficient for the generation of interstate coherence. This will be further described in Sec. IV in the context of nonlinear structural optical crystallography applications.

## IV. ULTRAFAST STRUCTURAL OPTICAL CRYSTALLOGRAPHY OF ELECTRONIC AND NUCLEAR DYNAMICS

Electronic structure dynamics are the origin of the nuclear structural change, and structurally sensitive measurements directly connect to those performed for nuclear dynamics. The study of electron correlation and X-ray spectroscopy covers important areas of ultrafast science which have seen many recent developments^27^ and are not explicitly considered here. Ultrafast coherent nonlinear spectroscopy of valence electron dynamics has emerged as a prominent technique to interrogate biological systems, notably photosynthetic dynamics.^97–102^ The coherence analysis and frequency-frequency correlation are accessible through such techniques which carry structural dynamics information indirectly. In order to add structural sensitivity of these observations, single crystal experiments are being developed, described further below. A real-space observation of electron density modification on ultrafast time scales would be a fascinating and complementary observation. Perhaps, a future experiment would measure the photoinduced electronic structure differences in addition to the nuclear response by ultrafast protein X-ray crystallography of protein crystals. In order to achieve this, orders of magnitude of signal-to-noise enhancement would need to be achieved in addition to subatomic resolution diffraction. Furthermore, since the full atomic cross section is measured by X-ray diffraction, the difference density resulting from displacement would overpower the valence electron density differences, and a separation of both signals would need to be carried out analytically. A separation of both responses in the time domain would perform such an experiment with attosecond resolution before Franck-Condon motion is initiated. For example, an attosecond experiment of a photosynthetic reaction center with weak excitation would measure the initial exciton formation, while femtosecond and picosecond delays could potentially detect the energy transfer reactions by using the nuclear displacements as a proxy of electronic excitation. Such experimental possibilities are, however, far removed from current capabilities. Valence electron dynamics need spectroscopic probes.

A structural sensitivity for spectroscopic observables exploits polarization and coordinate analysis from knowledge of the transition dipole direction. Traditionally, photoselection methods can provide such information. Either a linear or nonlinear spectroscopic measurement of photoselection, typically from isotropic materials and using polarized sources, generates structurally sensitive information which is essentially internal coordinates and specifically the dependence on the angles between transition dipole moments. Alternatively, polarized measurements of molecular crystals may provide more direct structural information as these are sensitive to crystal symmetry, the crystal laboratory orientation and index, and molecular structure.^39,40,96,103^ For a linear response analysis, molecular information may be obtained by including a coordinate analysis from X-ray crystallography with pleochroism, which is a subfield of optical crystallography.^104,105^ The propagation of light in crystals and anisotropic media has been established starting with the Fresnel equations two centuries ago.^104–109^ By combining knowledge from X-ray crystallographic coordinates and associated transition dipole gradients with the ensemble averaged field-dipole correlation of a given rank which becomes the sum of crystallographic symmetry progressions, a structural sensitivity results that can be directly visualized in the molecular basis.^40^

**FIG. 5.**
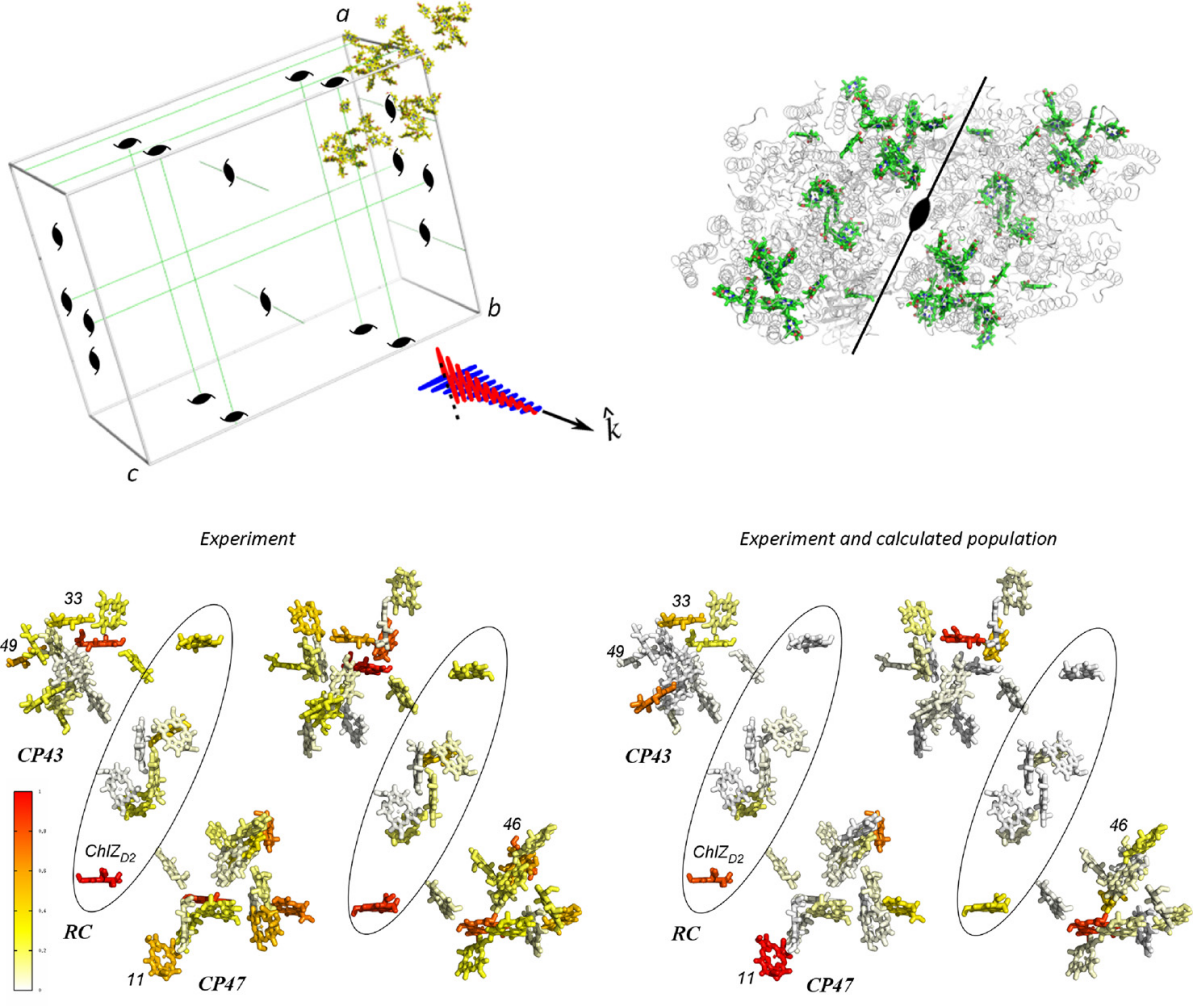
Polarized femtosecond mid-infrared measurements of oriented single orthorhombic microcrystals of *S. elongatus* Photosystem II core complexes.^39^ The linear response with the second rank is invariant for each and every symmetry operation in the unit cell, such that only the asymmetric unit can be analyzed to represent the unit cell response which includes a noncrystallographic twofold symmetry from the presence of a dimer complex. The experimental distribution (left) shows the projected projection amplitudes for excited state induced mid-infrared absorption at selected frequency at zero time. The right distribution adds the structure based calculation of the initial exciton generation in the single pigment basis to evaluate experimental amplitude and theoretical calculation. (Inset) Reproduced with permission from Kaucikas *et al.*, Nat. Commun. **7**, 13977 (2016). Copyright 2016 Nature.^39^

A reported application of ultrafast structural optical crystallography retrieved structural information from the combined use of crystallographic coordinates and optical crystallography.^39,103^ Polarized femtosecond mid-infrared measurements of oriented single microcrystals of *Synechococcus elongatus* Photosystem II core complexes measured the energy transfer and charge separation dynamics with structural information (Fig. 5). Photosynthetic energy transfer and charge separation are excellent examples of biological valence electron dynamics. The mid-infrared femtosecond measurement of optical excitation was chosen because this provides information in the single pigment basis, in contrast to the visible transient absorption measurement which is in the exciton basis. Effectively, the local oscillators probed in the mid-infrared act as projectors of the electronic excitation.^39,103^

Beyond the linear response analysis of ultrafast dynamics described, an extension of ultrafast structural optical crystallography to four-wave mixing is of particular interest. Two-dimensional electronic (2DES) and infrared spectroscopy (2DIR) have emerged as very powerful techniques to interrogate molecular systems on the ultrafast time scale.^97,98^ In both 2DES and 2DIR, frequency-frequency correlation measurements retrieve the contributions to the total four-wave mixing response.^97,98,110,111^ A review of the 2D spectroscopy fields and background is beyond the scope of this discussion, but an illustration of the optical crystallographic variant will be very briefly given. As with the photoselection experiment with linear response analysis, it is well known that polarization of the four fields involved modifies the 2D measurement in a quantifiable way sensitive to internal coordinates.^110,112^ The general method to evaluate the crystallographic four wave mixing response makes use of the response function formalism. As with the isotropic case, each contributing response function is weighted by the ensemble average of the field dipole interaction to the fourth rank.^110,112^ The ensemble average of the dipole term is found for crystals according to the following equation:^40^

$$\langle {\left(\hat{\mu }\cdot \hat{e}\right)}^{h}\rangle =\frac{1}{n}\sum_{k=1}^{n}{\left(\tilde{O_{k}}\cdot \hat{\mu }\cdot \hat{e}\right)}^{h},$$ (10)

which applies the symmetry operator O_k_ with n-fold symmetry and a rank of h for the number of interactions.

It has recently been shown that for all uniaxial and biaxial crystal classes, Eq. (10) evaluates the magnitude of all contributing response functions.^40^ Furthermore, the same method also allows an evaluation of the nonzero and zero-valued third order tensor elements, when written on the basis of the principal susceptibility directions and provides the zero and nonzero third order tensor elements directly for all crystal classes.^40^ It should be noted that additional symmetry elements present in the 32 point group symmetries that are in addition to those in the 7 crystal classes specify further selections for zero-valued third order tensor elements. The method is alternative to traditional techniques that use “direct inspection” involving coordinate transformations, such as tabulated by Boyd,^108^ Zernike and Midwinter,^113^and Shang and Shu.^114^

**FIG. 6.**
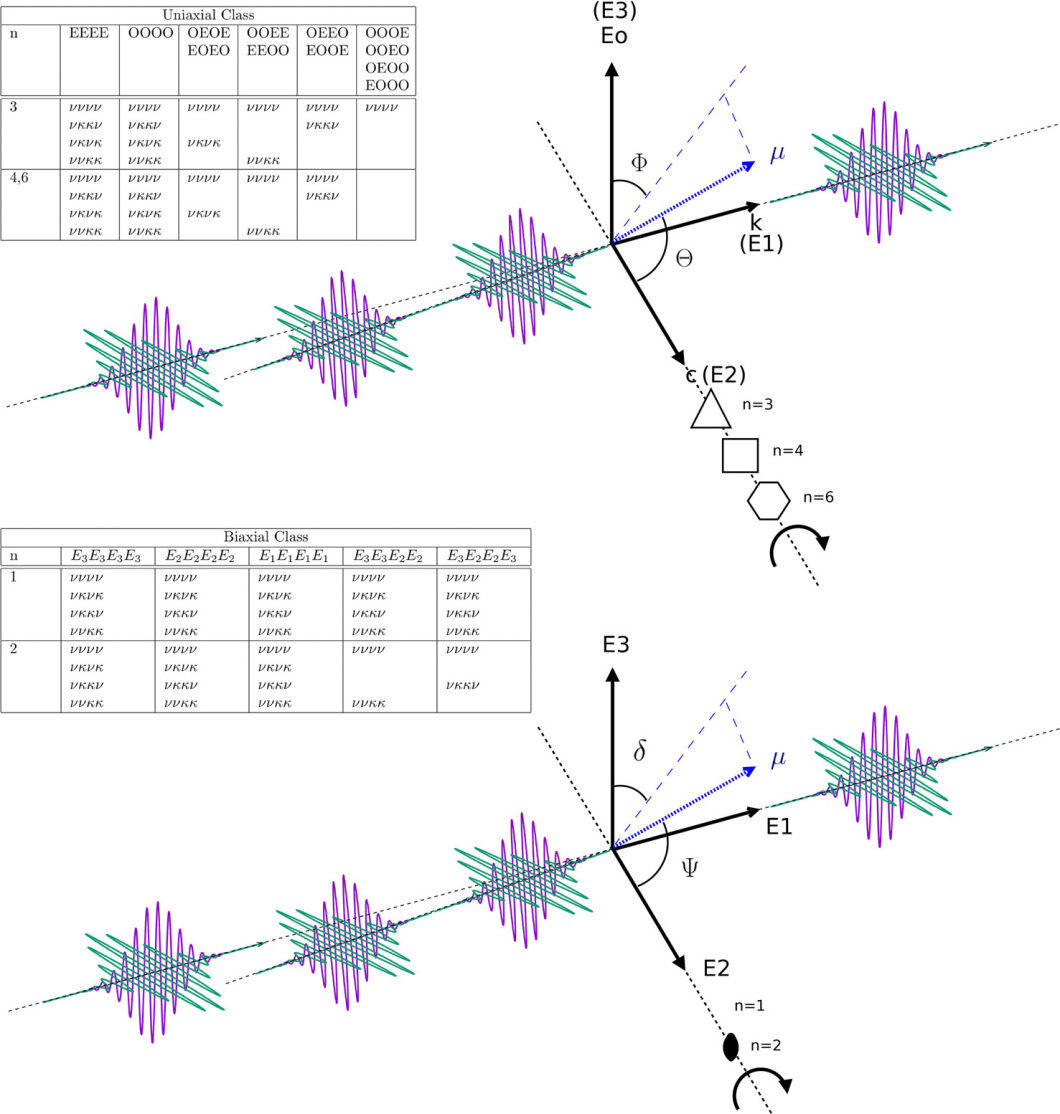
Allowed general quantum paths for two coupled oscillators ν and κ in uniaxial and biaxial crystal classes with reduced point group elements involving n-fold axes. The phase-matching geometry represented in the “pump-probe” geometry includes both the rephasing and nonrephasing third order signals. The table shows the allowed dipole combinations for polarization combinations in the tables, for which the values calculated via Eq. (10) have been presented previously.^40^ Note that allowed ν–κ permutations are implicitly included.

**FIG. 7.**
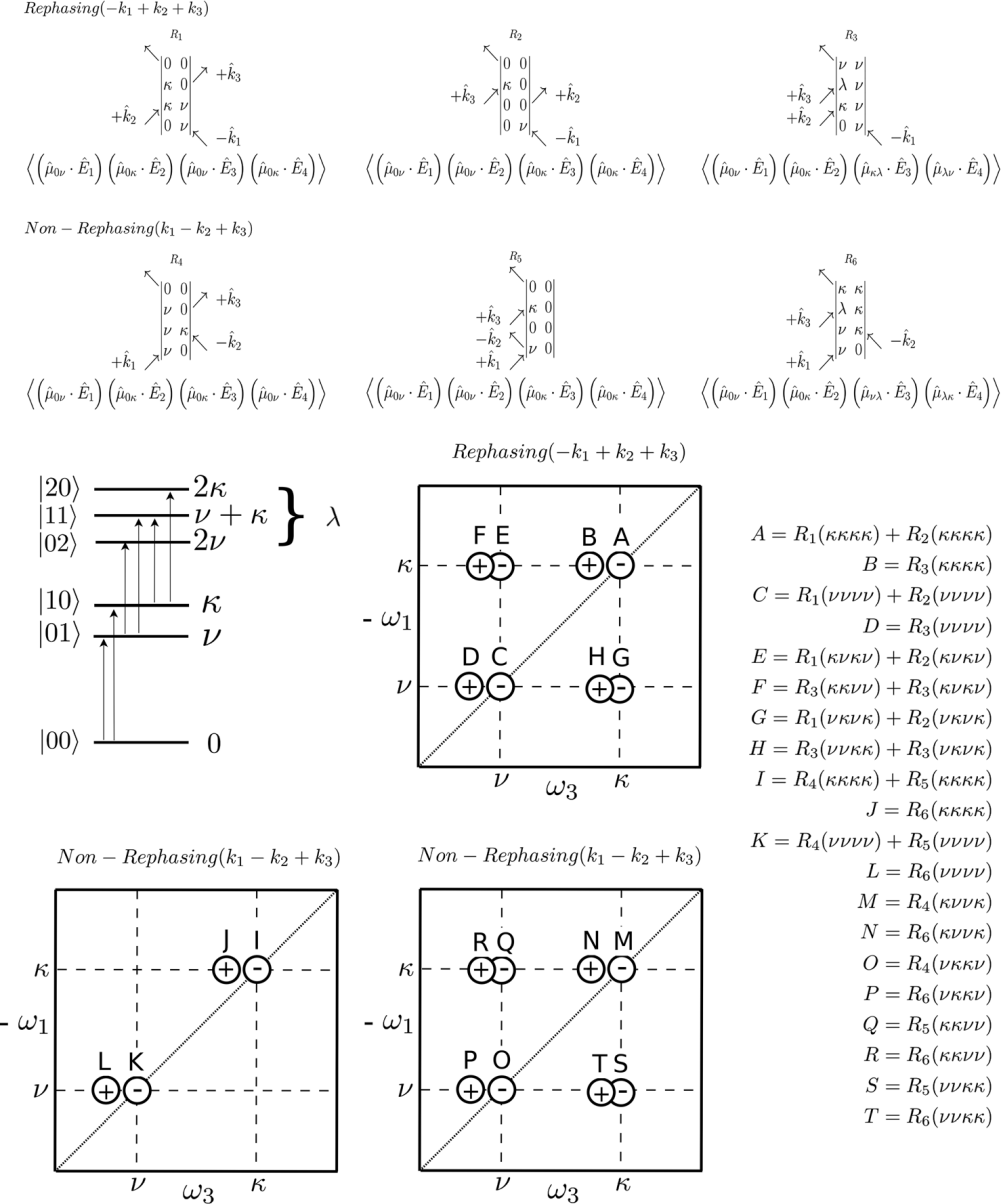
The general types of Feynman diagrams for two coupled oscillators ν and κ, shown for the rephasing and nonrephasing phase-matching conditions, represent the response function formalism. The (A, B, C, D, I, J, K, and L) diagonal and (E, F, H, G, M, N, O, P, Q, R, S, and T) cross peaks in the two-dimensional measurements are labeled according to the contributing response function(s) and Feynman paths. (Top of figure) Reproduced with permission from van Thor, Chem. Phys. **150**, 124113 (2019)]. Copyright 2019 AIP Publishing.^40^

It was further shown from explicit application of Eq. (10) for the case of coupled oscillators that particular Liouville pathways become symmetry disallowed in the presence of crystal symmetry, which is a similar condition to the zero-valued third order tensor elements for the isolated dipole arising in various point group symmetries (Fig. 6). For those Liouville pathways that are symmetry allowed, the magnitude of their coherence amplitudes has been calculated.^40^ Figure 6 shows a frame of reference for polarization directions in a possible phase-matching geometry that contains both the rephasing (k_s_ = –k_1_ + k_2_ + k_3_) and nonrephasing (k_s_ = k_1_ – k_2_ + k_3_) conditions for uniaxial and biaxial cases. The included tables show the allowed Feynman paths for a case of two coupled oscillators. From the absence in these included tables, it is further indicated which paths are disallowed for specific polarization conditions, such that unusual intensities for diagonal or cross peaks can develop. Previous work presented the calculation of the magnitudes of the allowed entries in the tables, which are not included here.^40^ These results also made possible an explicit simulation for 2D spectra of example orientations of sets of coupled oscillators using the response function formalism (Fig. 10 in Ref. 40). Figure 7 shows a specific overview of the types of Feynman paths, and the corresponding response functions give rise to which signals in a 2D measurement. These are valid for all 2D measurements that satisfy the coupling requirement and have sufficient separation of the natural frequencies (Fig. 7). The figure is given to illustrate the general result that specific diagonal or cross peaks can be eliminated through symmetry selection if these are disallowed for optical crystallography (Fig. 6) or through orientational selection if a field-dipole interaction is very small and suppresses the magnitude of the total four-point correlation function. The resulting conclusion is that the oriented single crystal acts as a “structural filter,” selecting and weighing the coherences that contribute to the 2D measurement.

For example, in trigonal symmetry, it is shown that for a case of two coupled oscillators ν and κ, with three interactions in the ordinary direction and a single extraordinary interaction, the only dipole allowed Feynman paths are νννν and κκκκ and all cross peaks are disallowed (Fig. 6, top). Inspection of Fig. 7 shows that for the rephasing directions, only diagonal signals remain, and their magnitudes are determined from the corresponding dipole terms by expanding Eq. (10) with the relevant point group symmetry according to Neumann's principle.^40^ For the nonrephasing phase matching condition, the situation appears to be similar, except that in addition to disallowing all cross peaks, also the diagonal peaks M,N,O and P are disallowed as these are also cross peaks yet appear on the diagonal. Therefore, their magnitudes will be additionally suppressed (Fig. 7).^40^ Next, considering the ⟨OEOE⟩ or ⟨EOEO⟩ polarization combination for trigonal symmetry, Figs. 6 and 7 illustrate that νννν, κκκκ, νκνκ, and κνκν pathways are allowed, whereas νκκν, κννκ, ννκκ and κκνν pathways are disallowed. As a result, diagonal peaks A, B, C, D, I, J, and K and L are nonzero depending on dipole orientation, whereas M, N, O, and P are fully disallowed from symmetry considerations. Similarly, cross peaks F and H only have contributions from R_3_(νκνκ) and R_3_(κνκν), but the nonrephasing cross peaks Q, R, S, and T and diagonal peaks M,N,O, and P are again disallowed (Fig. 7). The above illustrates that, in contrast to four-wave mixing measurements of isotropic samples where polarization may only modify the magnitude,^110,112^ crystal symmetry can fully disallow specific two dimensional signals. Furthermore, by analysis of the polarization combinations and resulting magnitudes of allowed response functions, the four-wave mixing signals carry direct structural sensitivity in the laboratory frame.^40^

As demonstrated previously with the femtosecond mid-infrared structural crystallography of photosynthesis,^39,103^ a coordinate based analysis needs rotation of the relevant dipole gradients onto the structure in the asymmetric unit followed by amplitude analysis from the point correlation functions. The implication is that many different methods of analysis of 2D spectroscopy can be analyzed with structural sensitivity. For example, the photosynthesis field has focused on oscillations seen in 2DES experiments during the waiting time t_2_, during which the system resides in an interstate coherence.^99–102,115^ Feynman diagrams contributing to the cross peaks (Fig. 7) are not in a population state during t_2_, but instead the density matrix resides in an interstate coherence ρ = |ν⟩⟨κ|. The phasors for the response functions show that during this time, the cross peaks will oscillate with the difference frequency between the modes ν and κ during t_2_. For 2DES measurements of photosynthesis, this feature has been analyzed and interpreted very differently by different authors, invoking either pure electronic coherence^101^ or electron-vibration coupling.^100^ Many currently agree that electron-vibration coupling is the most likely assignment, but the proposed mechanisms differ. Tiwari *et al.* made a compelling case for assignment to nonadiabatic electron-vibration coupling.^100^ The quantum beating during waiting time would present a very interesting example to address with 2D structural optical crystallography. Practically, challenges will involve the experimental execution. It requires knowledge of laboratory orientation of micrometer sized crystals, which must be obtained by X-ray crystallographic face indexing if the crystal symmetry is biaxial and birefringence analysis is ambiguous. Such was the case with orthorhombic crystals of *S. elongatus* PSII, which required time-delayed imaging of crystal growth morphology and extensive statistics from face indexing of larger crystals.^39,103^ Additional experimental challenges including phase-matching have been discussed.^40^ This is, however, only one possible application. The four-wave mixing application of structural optical crystallography has been proposed recently together with methods for the directional retrieval of coherence amplitudes. This could have many applications in different areas including energy science.

## V. CONCLUDING REMARKS

This perspective aims to discuss together the different structural aspects of molecular processes that are detected in X-ray crystallographic and ultrafast and nonlinear structural optical crystallography. A snapshot of developments in the different related areas of ultrafast structural dynamics is given with an emphasis on protein dynamics and single crystal applications. While a brief overview of some history of X-ray methodology and ultrafast X-ray sources has been included, the focus here is on formulating open questions and opportunities in molecular physics that naturally arise from the fast developing fields. The ability to measure ultrafast X-ray crystal structures of proteins is only a few years old, but it can mine and exploit decades of ultrafast spectroscopy and fundamental theory developed in this field. The present contribution probably only covers a small part of this, but it has been presented with practical applications for analysis in mind. Here, the primary questions of interest are specific to femtosecond dynamics and nuclear coherence that can be addressed using methods taken from ultrafast spectroscopy. Similarly, the multiphoton processes that are practically unavoidable in order to achieve detectable photoinduced differences can and must be quantified and controlled. When structural measurements of electronic coherence and dynamics are of interest, the structural optical crystallography method can provide such information directly in the molecular frame, and methods for evaluating contributing coherence amplitudes to four-wave-mixing have been described. The intention of this perspective was to discuss future opportunities for both areas of structural dynamics. In practice, the successful execution of both techniques relies on applying crystal optics such that the molecular dynamics are understood and measured by spectroscopy techniques with structural sensitivity. The two specific methods that have been discussed, ultrafast X-ray crystallography and ultrafast nonlinear structural optical crystallography, will continue to develop, benefit from, and rely upon each other.
